# Investigation of the Effects of Electrode Geometry on the Performance of C^4^D Sensor with Radial Configuration

**DOI:** 10.3390/s21134454

**Published:** 2021-06-29

**Authors:** Qiang Huang, Junchao Huang, Yandan Jiang, Haifeng Ji, Baoliang Wang, Zhiyao Huang

**Affiliations:** State Key Laboratory of Industrial Control Technology, College of Control Science and Engineering, Zhejiang University, Hangzhou 310027, China; huang_q@zju.edu.cn (Q.H.); ydjiang@zju.edu.cn (Y.J.); hfji@zju.edu.cn (H.J.); wangbl@zju.edu.cn (B.W.); zy_huang@zju.edu.cn (Z.H.)

**Keywords:** capacitively coupled contactless conductivity detection, electrode geometry, radial configuration, conductivity measurement

## Abstract

Electrodes are basic components of C^4^D (capacitively coupled contactless conductivity detection) sensors, and different electrode structures (the configuration pattern or the electrode geometry) can lead to different measurement results. In this work, the effects of electrode geometry of radial configuration on the measurement performance of C^4^D sensors are investigated. Two geometrical parameters, the electrode length and the electrode angle, are considered. A FEM (finite element method) model based on the C^4^D method is developed. With the FEM model, corresponding simulation results of conductivity measurement with different electrode geometry are obtained. Meanwhile, practical experiments of conductivity measurement are also conducted. According to the simulation results and experimental results, the optimal electrode geometry of the C^4^D sensor with radial configuration is discussed and proposed. The recommended electrode length is 5–10 times of the pipe inner diameter and the recommended electrode angle is 120–160°.

## 1. Introduction

**Figure 1:**
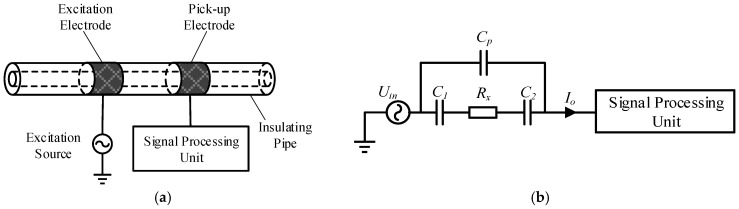
C^4^D sensor: (**a**) The typical construction; (**b**) The equivalent circuit.

The capacitively coupled contactless conductivity detection (C^4^D) method is an effective conductivity detection method for fluidic analysis and measurement [1,2,3,4]. Compared with traditional contact conductivity detection methods, the electrodes of the C^4^D sensor are not in direct contact with the measured fluid. Hence, some unfavorable influence of contact conductivity detection methods (such as the electrode polarization and the electrochemical corrosion) can be avoided [1,2,3,4,5]. Due to its advantages, the C^4^D method has attracted many researchers’ attention and made considerable development since it was proposed [1,2,3,4]. Figure 1a illustrates the typical construction of a C^4^D sensor. As illustrated, a C^4^D sensor consists of an AC source, an excitation electrode, a pick-up electrode, an insulating pipe and a signal process unit. The excitation electrode and pick-up electrode, which are connected with the excitation source and the signal process unit respectively, are attached to the outer wall of the pipe. Figure 1b shows the equivalent circuit of the C^4^D sensor, where $R_{x}$ represents the equivalent resistance of the fluid, $C_{1}$ and $C_{2}$ represent the coupling capacitances and $C_{p}$ represents the stray capacitance. When an AC voltage is applied on the excitation electrode, the current is measured on the pick-up electrode, and then the conductance of the fluid between the two electrodes can be obtained.

**Figure 2:**
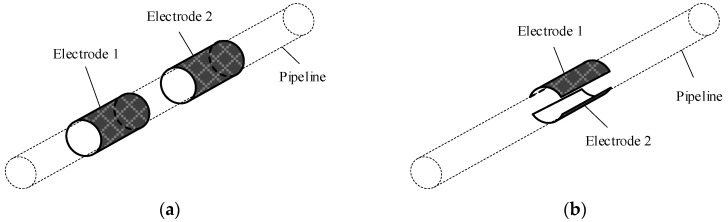
The C^4^D sensor with different electrode configurations: (**a**) Tubular configuration; (**b**) Radial configuration. Notation: For the tubular configuration (as illustrated in (**a**)), a pair of cylindrical electrodes is attached around the pipe. For the radial configuration (as illustrated in (**b**)), a pair of concave electrodes is attached to the pipe oppositely.

There are two kinds of electrode configuration patterns of C^4^D sensor, the tubular configuration and the radial configuration, as illustrated in Figure 2 [1,4,6,7,8]. The tubular configuration is the most commonly used electrode structure of C^4^D sensor. For this configuration, the fluid is measured in the axial direction of the pipeline. The sensor with tubular electrodes can obtain a large and stable signal, which helps the improvement of detection performance [6,9]. Through decades of sustained development, C^4^D sensors have been studied by many researchers and are mostly employed for detection in capillary electrophoresis (CE) and microchip electrophoresis (MCE) (the inner diameter of the pipe is always less than 0.5 mm) [1,2,3,4,10]. In these fields, the tubular configuration can meet the measurement requirement well. Therefore, most of the published works focus on the application of the tubular configuration, while there are few on the radial configuration. Recently, the potential of applying C^4^D to non-electrophoretic fields has been mined and the pipe scale has been extended beyond the original capillary or microchannel (the inner diameter of the pipe is larger than 1 mm) [1,5,9,11,12,13]. To realize satisfactory measurement performance, both the ratio of the tubular electrode length to the pipe diameter and the ratio of the tubular electrodes distance to the pipe diameter are usually large enough (larger than 5 or even 10) [2,3,4,9,14,15]. The ratios are acceptable for pipes with smaller inner diameters. However, with the increase of the pipe diameters, the ratios will result in large electrodes and large sensor sizes, which may cause inconvenience in arranging and applications. This is one of the reasons why the research works and the applications of C^4^D sensors with the tubular configuration are limited in the pipes with larger inner diameters [1,4,5,6,9,16].

Radial configuration provides another effective application for C^4^D. Radial configuration is more compact than tubular configuration so it is applicable for normal-scale (millimeter or tens of millimeter scale) pipes and can avoid the aforementioned defects of the tubular configuration [5,9,17]. Furthermore, for sensors with radial configuration, the fluid is measured in the radial direction of the pipeline. This characteristic is different from the tubular configuration and means the detection areas and the obtained information of the two configurations are different. The detection area of the tubular configuration is the pipe area between the two electrodes and the obtained information is the average conductivity of a section of fluid in the pipe, while for the radial configuration, the detection area is the pipe area covered by the two electrodes, and the obtained information is the fluid conductivity in the cross-section of the pipe and embodies the features of the fluid cross-section. Despite these advantages, the radial configuration pattern receives little attention in the research field of C^4^D [3,5,6,17], and is usually adopted in the research works on capacitance sensors [18,19,20,21]. Accordingly, to exploit the potentials of radial configuration, more relevant research should be undertaken.

Researchers have made great efforts to the design of C^4^D sensors, such as optimization of the sensor structure [1,2,3,14,15,17,22] and improvement of the measurement circuit [1,7,16,23,24,25]. The selection of the electrode structure, i.e., the configuration pattern and the geometry (geometrical parameters) is a key point [1,2,3,9,14,15,17,18,22,26]. The design of electrodes is a basic process for detection and an attractive research aspect for researchers [14,15,17,22]. For a certain configuration pattern, the electrical field distributions are determined by the geometry of electrodes. Therefore, the geometry of the electrodes can affect the response of the C^4^D sensor, and hence affect the measurement result. Unfortunately, the research works on the design of the electrode geometry for radial configuration are limited, and the geometry can only be designed empirically. In order to fill this technique gap, the electrode geometry of radial configuration needs more research works.

The effects of electrode geometry design of radial configuration on the measurement performance of the C^4^D sensor are investigated in this work. Both simulation analysis and practical experiments are carried out. The length and the angle of electrodes are considered. A simulation model of the C^4^D sensor with radial configuration is developed and used for the conductivity measurement. The relationship between the conductivity and the output of the C^4^D sensor is acquired. With different electrode geometry, different measurement results are obtained. According to these simulation results, the measurement performance of the C^4^D sensor is analyzed and contrasted. A group of C^4^D sensors with different electrode geometries are fabricated and conductivity measurement experiments are conducted. Then, comparison between the simulation results and the practical experimental results is carried out. Finally, the optimal electrode geometry of radial configuration is discussed and proposed according to the research results.

## 2. Simulation

**Figure 3:**
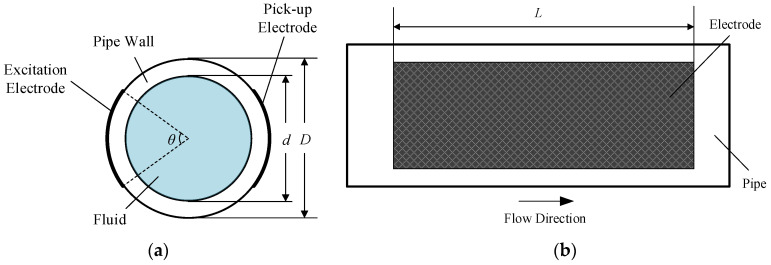
Views of the radial configuration: (**a**) The sectional view; (**b**) The lateral view.

The sectional view and lateral view of the C^4^D sensor with radial configuration are illustrated in Figure 3, where *d* represents the inner diameter of the pipe, *D* represents the outer diameter of the pipe, *θ* represents the angle of the electrodes and *L* represents the length of the electrodes. The two electrodes are placed right against one another. Obviously, the geometry of the electrodes depends on the electrode length *L* and the electrode angle *θ*. To investigate the effects of the electrode geometry on the measurement performance of the C^4^D sensor, simulation analysis is carried out.

### 2.1. FEM Model of the C^4^D Sensor

A 3D model based on the finite element method (FEM) is developed to study the electrical field and hence to calculate the conductance of the measured fluid. The model is developed and calculated by the software COMSOL Multiphysics 5.5 (COMSOL Inc., Stockholm, Sweden). Electrical field interface is used and the problem is to solve the equation of continuity, which can be expressed in the frequency domain as:

(1) $$\nabla \cdot \left(\left(\sigma \left(x,y,z\right)+j2\pi f\varepsilon_{0}\varepsilon_{r}\left(x,y,z\right)\right)\nabla \varphi \left(x,y,z\right)\right)=0,\;\;\left(x,y,z\right)\in \Pi$$

where σ(x,y,z), $\varepsilon_{r}\left(x,y,z\right)$ and φ(x,y,z) are the distribution functions of the conductivity, relative permittivity and electrical potential respectively, $\varepsilon_{0}$ is the permittivity of vacuum, f is the excitation frequency, *j* is the imaginary unit, ∇ is the Hamiltonian operator and Π is the model domain. In this equation, φ(x,y,z) is the dependent variable.

In the C^4^D sensor, one of the electrodes is connected with the excitation source, and the other is connected with the virtual ground. Therefore, the potentials of the excitation electrode and the pick-up electrode in the FEM model are set as constants $\varphi_{0}$ and 0, respectively. This can be expressed as:

(2) $$\varphi \left(x,y,z\right)=\varphi_{0},\;\left(x,y,z\right)\in \Gamma_{1}$$

(3) $$\varphi \left(x,y,z\right)=0,\;\left(x,y,z\right)\in \Gamma_{2}$$

where $\Gamma_{1}$ and $\Gamma_{2}$ are the boundaries of the excitation electrode and the pick-up electrode, respectively.

Because of the existence of the reactive component, the impedance (including both real part and imaginary part) is actually calculated in the model. While the sensor is working, the current passing through the pick-up electrode can be calculated:

(4) $$I=\int_{\Gamma_{2}}Jd\Gamma_{2}$$

where J is the conduction current density and J=−σ∇φ. Then, the impedance can be calculated by Ohm’s law:

(5) $$Z_{x}=\frac{\varphi_{0}-0}{I}=R_{x}+jX_{x}$$

where $R_{x}$ and $X_{x}$ present the real part and the imaginary part of the impedance, respectively.

**Figure 4:**
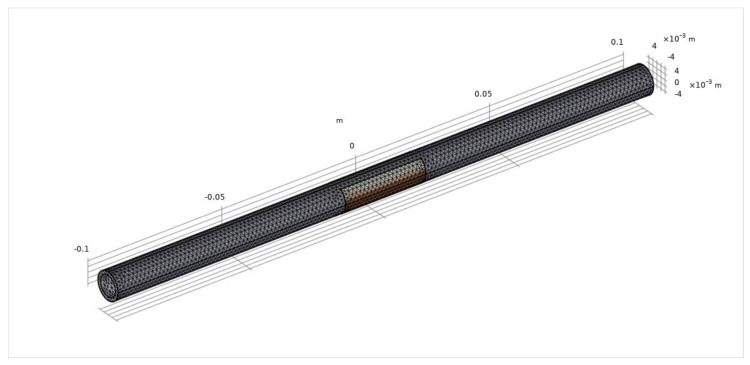
The main domain of the FEM model.

**Figure 5:**
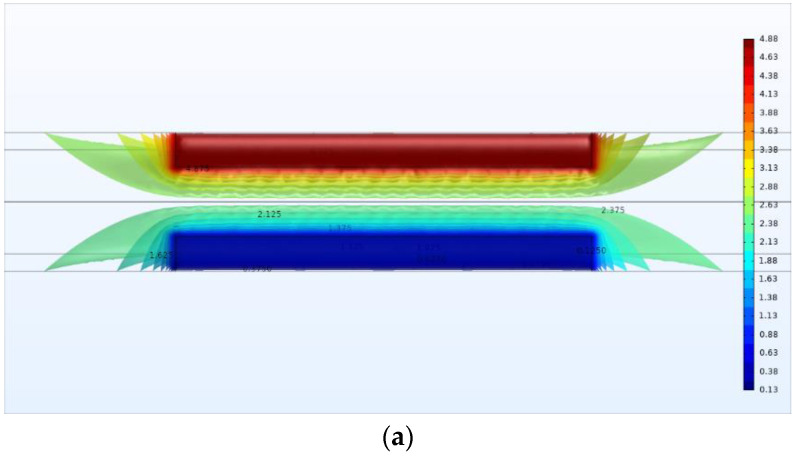

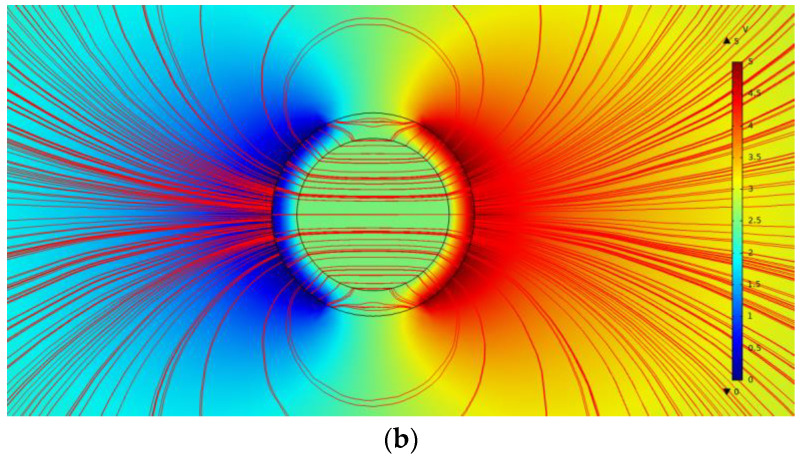
The FEM model: (**a**) The top view of the distribution of the electrical potential; (**b**) The sectional view of the distributions of the electrical potential and the electric field lines.

The developed FEM model consists of the pipe, the C^4^D sensor and the external environment. The main domain of the model is shown in Figure 4. The external environment is full of air. The inner diameter *d* and outer diameter *D* of the pipe are 7.5 mm and 10 mm, respectively. In the pipeline, the solutions with conductivity ranging from 0.04 mS/cm to 10 mS/cm are simulated. Table 1 lists the properties of the model components. The property of the pipe wall is set to be similar to quartz glass, i.e., the conductivity is 0 and the relative permittivity is 4.2. There are two electrodes attached to the outer wall of the pipe and the material of the electrodes is set as copper. One of the electrodes is supplied with the excitation voltage and the other is grounded. The amplitude of the excitation voltage is set as 5 V and the determination of the frequency is separately discussed in the later sections. The conductivity and relative permittivity of the external air are 0 and 1, respectively. The relative permittivity of the solutions is 78. Tetrahedral quadratic elements are used as the mesh mode of the FEM model. Figure 5 shows the distributions of the electrical potential and the electric field around the electrodes from different views.

**Figure 6:**
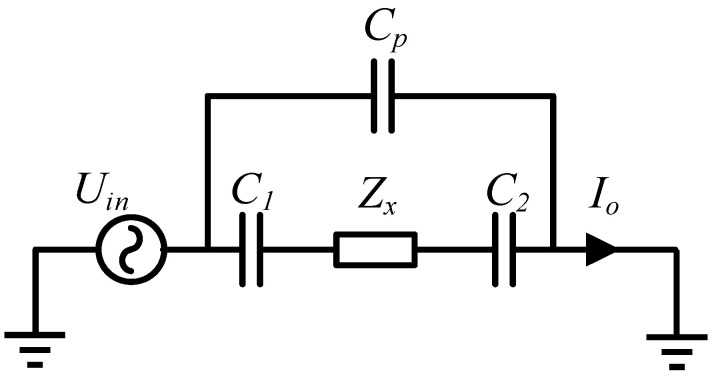
The equivalent circuit in the simulation analysis.

As shown in Figure 5b, besides the area of the detection pipe, the electric field lines are also distributed in the external air and pass through the external air from the excitation electrode to the pick-up electrode. These electric field lines can be regarded as a stray capacitance in parallel with the detection path. Thus, the equivalent circuit in the simulation analysis can be illustrated as Figure 6, where $C_{p}$ represents the stray capacitance.

### 2.2. Simulation Results

#### 2.2.1. Effects of the Excitation Frequency

**Figure 7:**
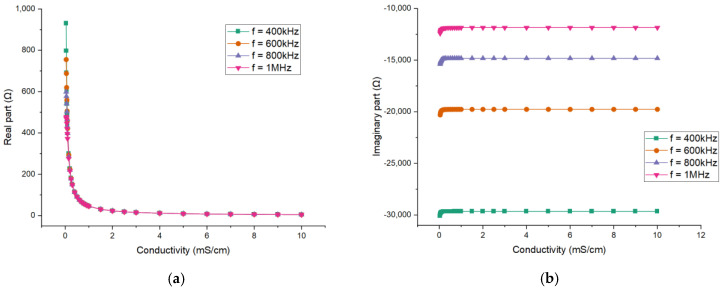
Simulation results for different excitation frequencies: The dependence of (**a**) the real part of the impedance; (**b**) the imaginary part of the impedance.

The choice of the excitation frequency in the simulation deserves consideration. The excitation frequency will affect the value of the obtained impedance. Therefore, the effects of the excitation frequency are investigated. With the FEM model, the real part and the imaginary part of the impedance can be obtained and the simulation results for different excitation frequencies are shown in Figure 7. The curves of 400 kHz, 600 kHz, 800 kHz and 1 MHz are respectively given.

The impedance results indicate that the absolute values of both parts will decrease when the conductivity increases. The absolute value of the imaginary part is much larger than that of the real part. Obviously, the imaginary part is mainly contributed by the coupling capacitance. Therefore, the imaginary part is nearly constant when the conductivity of the solution is high enough. The capacitive reactance of the solution will have effect when the conductivity is low enough, which leads to the slight variation in the imaginary part. The variation of conductivity is mainly reflected in the value of the real part. Therefore, the real part of the impedance is extracted for the measurement. Meanwhile, the relationship between the conductance $G_{x}$ and the conductivity σ is studied, and the conductance can be expressed as:

(6) $$G_{x}=\frac{1}{R_{x}}$$

**Figure 8:**
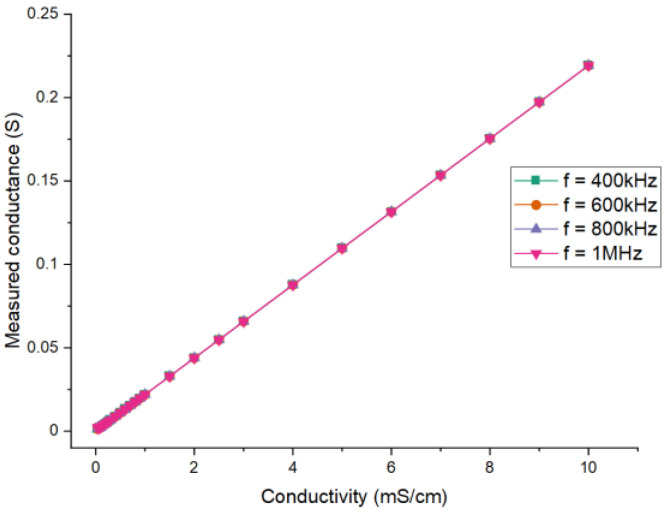
Simulation results for different excitation frequencies: the relationship between the measured conductance and the conductivity.

Figure 8 shows the relationship between the measured conductance $G_{x}$ and the conductivity σ for different excitation frequencies. From all the results, it can be seen that the differences in the imaginary part of the impedance between different frequencies are significant. The values of the real part (also conductance) between different frequencies are almost the same and their differences are mainly found at low conductivity. The differences result from the reactive component of the measured fluid, which cannot be neglected at low conductivity.

Corresponding research is also carried out by experiments, and the discussion can be found in Section 3.2.1. Based on the simulation and experimental results, 1 MHz is adopted as the excitation frequency in the simulation and in experimental research on the effects of the electrode length and angle.

#### 2.2.2. Effects of the Electrode Length

**Figure 9:**
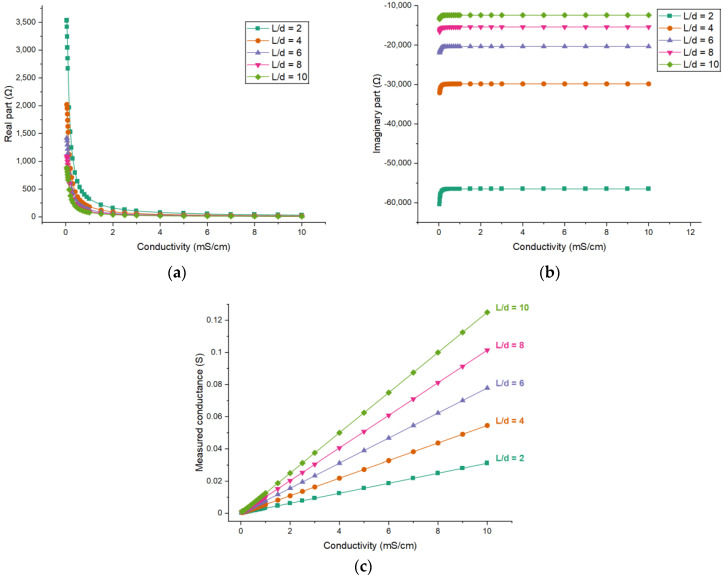
Simulation results for different electrode lengths: The dependence of (**a**) the real part of the impedance; (**b**) the imaginary part of the impedance; (**c**) the measured conductance on the conductivity of the solution when *θ* = 120°.

The electrode length of the radial configuration (i.e., the *L* shown in Figure 3b) determines the size of the detection domain along the axis direction of the pipe. With the developed FEM model, the effects of the electrode length on the measurement performance of C^4^D sensor are investigated. By changing the electrode length in the FEM model, different measurement results can be obtained. The curves describing the relationships between each part of the measured impedance and the conductivity of the solution are diverse. As such, the real part and the imaginary part of the impedance are investigated respectively. Figure 9 shows the simulation results for different electrode lengths (taking the inner diameter of the pipe *d* as the reference, setting *L/d* = 2, 4, 6, 8 and 10) when the electrode angle *θ* is fixed to 120°.

Figure 9c shows the relationship between the measured conductance and the conductivity for different electrode lengths. It can be seen that the measured conductance is nearly linear with the conductivity of the solution, and with the increase of the electrode length the sensitivity (i.e., $\frac{\Delta G_{x}}{\Delta \sigma }$) of the measurement increases.

#### 2.2.3. Effects of the Electrode Angle

Because the two electrodes are placed opposite each other for the radial configuration, the electrode angle of the radial configuration (i.e., the *θ* shown in Figure 3a) can also be regarded as the gap width between the two electrodes, and the larger the angle, the shorter the gap width. Therefore, the electrode angle is also a main parameter that can determine the detection domain. Similarly, the effects of the electrode angle on the measurement performance of the C^4^D sensor are investigated by the FEM model.

**Figure 10:**
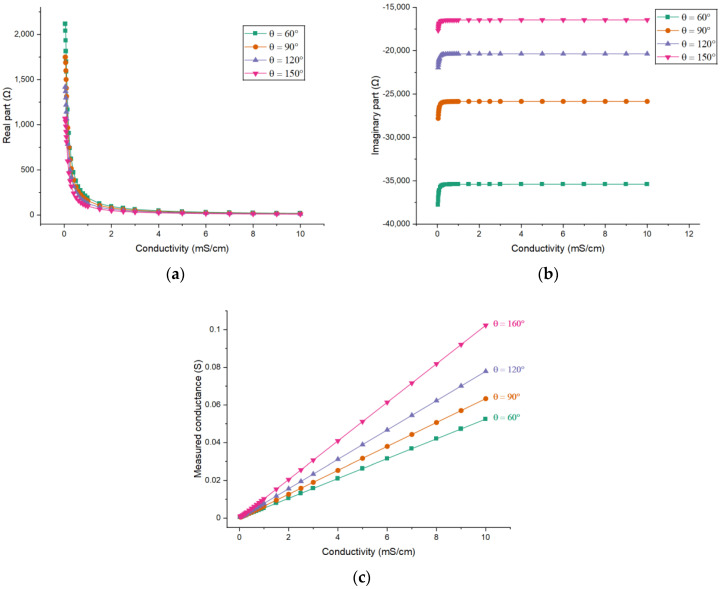
Simulation results for different electrode angles: The dependence of (**a**) the real part of the impedance; (**b**) the imaginary part of the impedance; (**c**) the measured conductance on the conductivity of the solution when *L/d* = 6.

The FEM model is used to obtain the simulation results for different electrode angles (60°, 90°, 120° and 150°). Figure 10 shows the simulation results when the electrode length *L* = 45 mm, i.e., *L/d* = 6.

Figure 10c shows the relationship between the measured conductance and the conductivity for different electrode angles. All relationships are also nearly linear and with the increase of the electrode angle the sensitivity of the measurement will also increase. Further, the improvement of sensitivity by increasing the electrode angle will be greater when the angle is larger.

## 3. Experiments

### 3.1. Experimental Setup

To validate the effectiveness of the simulation results, practical experiments were carried out. A group of C^4^D sensors with radial configuration were fabricated. The copper foils (the thickness is 0.30 mm) are used as the electrodes and contact of the electrodes with the pipe wall is realized by an acrylic modified epoxy adhesive. Table 2 lists the geometry parameters of all the C^4^D sensors used in the experiments.

**Figure 11:**
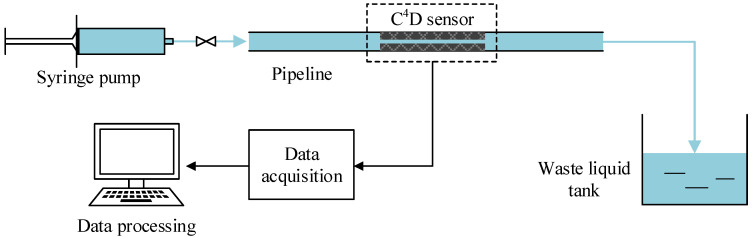
The layout of the experimental setup.

With these sensors, conductivity measurement experiments were conducted. The relationship between the output of the C^4^D sensor and the conductivity of the measured solution was obtained. The layout of the experimental setup is illustrated in Figure 11, which consists of a syringe pump, a C^4^D sensor and a data acquisition and processing unit. KCl solutions with different conductivities ranging from 0.04 mS/cm to 10.00 mS/cm were used as the measured fluid. The reference conductivity of the solution was measured by a commercial contact conductivity meter (FE38-Meter, Mettler Toledo Inc., Zurich, Switzerland). The KCl solutions were driven into the pipe by the syringe pump (Syringe Pump Model 33, HARWARD Apparatus Inc., Holliston, MA, USA). The inner diameter *d* and the outer diameter *D* of the insulating pipe were 7.76 mm and 10.26 mm, respectively. A signal generator (CA1640-02, RIGOL Technologies Inc., Suzhou, China) was used to generate the AC excitation source. The voltage of the excitation source was set as 10.0 Vpp. An analog phase sensitive demodulation (APSD) unit was introduced to obtain the total impedance (both the real part and the imaginary part), and more detailed information about APSD is available in [5]. The KCl solutions were prepared with deionized water, the conductivity of the deionized water was $5.5\times 10^{-5}$ mS/cm and the relative permittivity was around 78. The temperature during the experiments was about 22.0 °C.

### 3.2. Experimental Results

#### 3.2.1. Effects of the Excitation Frequency

**Figure 12:**
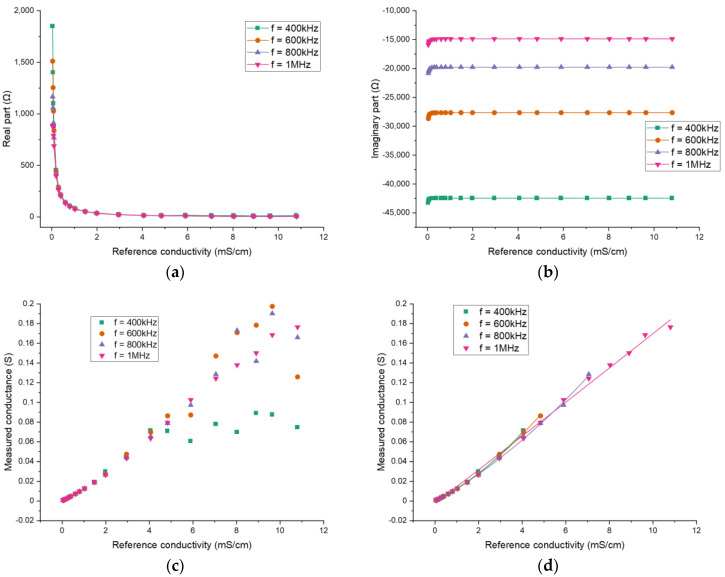
Experimental results for different excitation frequencies: The dependence of (**a**) the real part of the impedance; (**b**) the imaginary part of the impedance; (**c**) the measured conductance on the conductivity of the solution; (**d**) the conductance results with conductivity within the corresponding upper limit.

Similar to the simulation analysis, experiments were conducted to investigate the effects of the excitation frequency on the measurement and hence to determine the excitation frequency used in the following experimental study. Figure 12 shows the experimental results of conductivity measurement of KCl solution under different excitation frequencies (400 kHz, 600 kHz, 800 kHz and 1 MHz).

It can be seen that there exists significant difference in the values of imaginary part of the impedance for different frequencies. For the real part, the difference is minor. Figure 12c shows the results of measured conductance. As shown, the measurement performance deteriorates at high solution conductivity and there is an upper limit of the conductivity measurement. Furthermore, it can be found that the upper limit decreases with the decrease of the excitation frequency, which is unfavorable for the measurement of high conductivity. At high conductivity, the real part of the fluid impedance is too small and is covered by the imaginary part so the measurement is limited. A high excitation frequency can help alleviate this problem. Figure 12d shows the results with conductivity within the corresponding upper limit for different frequencies and the curves are also similar. Above all, a higher excitation frequency is suitable for the measurement in this conductivity range. In addition, to ensure a proper working frequency for the electronic components, 1 MHz is selected for the experiments.

#### 3.2.2. Effects of the Electrode Length

**Figure 13:**
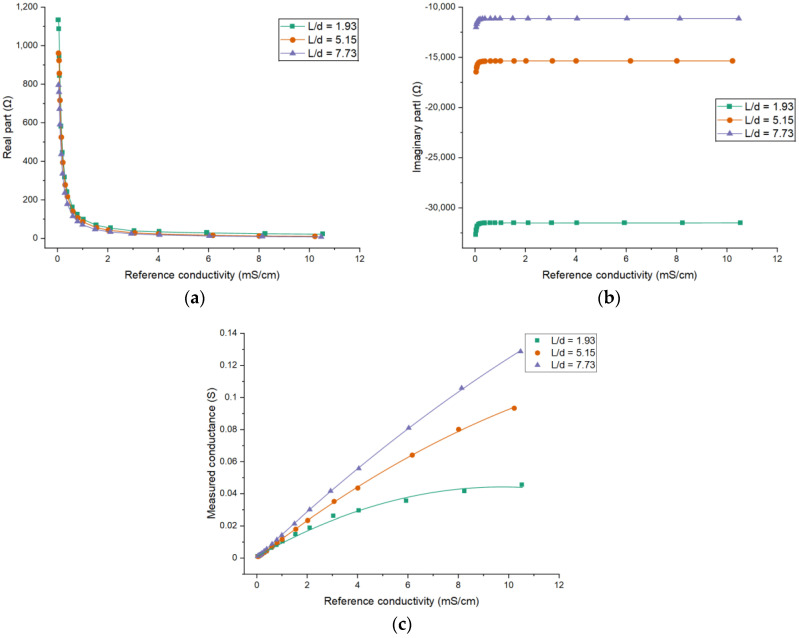
Experimental results for different electrode lengths: The dependence of (**a**) the real part of the impedance; (**b**) the imaginary part of the impedance; (**c**) the measured conductance on the conductivity of the solution when *θ* = 120°.

Sensors 1, 2 and 3 are used to investigate the effects of the electrode length. Figure 13 shows the experimental results. It can be seen that the absolute values of both parts of the impedance decrease with the increase of the conductivity of the solution. Furthermore, the absolute value of the imaginary part is much larger than that of the real part due to the existence of the coupling capacitances. For different electrode lengths, the obtained impedances are different. The larger electrode length results in smaller absolute values of both parts. The results are similar to the simulation results.

The relationship between the measured conductance and the reference conductivity for different electrode lengths is given in Figure 13c. According to the results, it can be found that the curves are different for different electrode lengths and the curve linearity for Ld=1.93 is worse than that of the other two curves. Meanwhile, comparing the results obtained from different electrode lengths, a larger electrode length can result in higher measurement sensitivity. This is also in accord with the simulation results.

#### 3.2.3. Effects of the Electrode Angle

**Figure 14:**
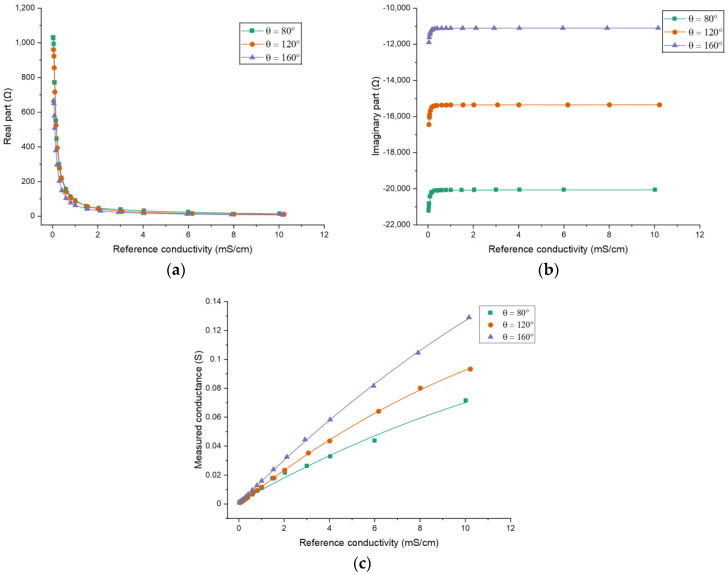
Experimental results for different electrode angles: The dependence of (**a**) the real part of the impedance; (**b**) the imaginary part of the impedance; (**c**) the measured conductance on the conductivity of the solution when *L/d* = 5.15.

Sensors 2, 4 and 5 are used to investigate the effects of the electrode angle. Figure 14 shows the experimental results. The experimental results are also similar with the simulation results. It can be seen that the larger electrode angle results in smaller absolute values of both parts of the impedance. Figure 14c shows the results on measured conductance. As shown, the sensitivity increases with the increase of the electrode angle.

## 4. Discussions

Generally speaking, the simulation results are in agreement with the experiment results. The measured conductance increases with the increase of the solution conductivity in both simulation and experiments for the C^4^D sensor with radial configuration. Considering different electrode geometry, the results of a larger electrode length or a larger electrode angle display higher sensitivity of conductance measurement according to both the simulations and the experiments.

According to the results, selecting a larger electrode length and a larger electrode angle could be the criterion to realize better measurement performance when designing the C^4^D sensor with radial configuration. However, a longer electrode means more occupation, which deviates from the advantages of the radial configuration. Therefore, the electrode length should not be too long. According to the results, the recommended electrode length is 5–10 times of the pipe inner diameter. For electrode angle, the upper limit is 180°. However, when it is set near 180°, some unpredictable influence from the external environment may emerge and the fabrication will be more difficult. As such, it is necessary to reserve a certain margin for electrode angle and we recommend an electrode angle of 120–160°.

It’s worth mentioning that there also exist slight differences between the simulation results and experimental results because of the difference between simulation and experiment and the non-ideal factors in the experiments. The relationship between the measured conductance and the conductivity obtained by simulation is strongly linear while the linearity of the relationship obtained by experiments is not good at high conductivity. This difference may result from the non-ideality of the practical experimental environment. The measured value of the real part is small and its variation to the conductivity is tiny at high conductivity. For practical experiments, the useful signal is small and will be covered by the noise, i.e., the SNR (signal to noise ratio) is low. For simulation, the noise is not considered. Further, the differences between the real parts of the simulation and the practical experiments are small, while for imaginary parts the differences are relatively large. In a C^4^D sensor with radial configuration, the imaginary part is determined by the coupling capacitances, the stray capacitance and the capacitive component of the measured fluid. The coupling capacitances are determined by the relative permittivity and the thickness of the pipe wall. In simulation, the thickness and the relative permittivity are fixed. In practical experiments, although the thickness and the relative permittivity of the pipe wall are almost same as the simulation, the external environment also influences the coupling capacitances. Additionally, the influence of the adhesive between the electrodes and the pipe on the coupling capacitances should also be considered. The above phenomenon also indicates that the C^4^D sensor with radial configuration has potential for measurement of permittivity [17,27]. Notably, some research has monitored the permittivity of the mixture of ethanol and gasoline, and hence implementing the determination of the ethanol content of gasoline with a sensor has a similar structure [27].

## 5. Conclusions

Radial configuration is one of the effective configurations for C^4^D sensors and few research works on it have been reported. Different from tubular configuration, radial configuration is more compact, because the electrode length and the electrode distance of tubular configuration are always set as 5–10 times of the inner pipe diameter. Thus, with the increase of the pipe diameter, the detection area of a tubular C^4^D sensor will be 15–30 times of the pipe inner diameter. For a radial C^4^D sensor, the detection area is 5–10 times the pipe inner diameter. Hence, it is suitable for the large pipe. Its geometry design is a significant research aspect. In this work, the effects of the electrode geometry on the performance of C^4^D sensors with radial configuration are investigated. Electrode length and electrode angle are the considered geometry parameters. The research is carried out by both simulation and experiment.

A FEM model based on the C^4^D method is developed to implement the simulation analysis. By this model, conductivity measurement is conducted with different electrode geometry. The relationship between the measured conductance and the reference conductivity is obtained. The relationship displays good linearity and indicates that the measured conductance increases with the increase of the conductivity. For different electrode geometry, the results are different. The effects of the electrode geometry on the measurement performance are analyzed and discussed by contrasting these results. It can be found that a larger electrode length or electrode angle will result in larger measured conductance and higher measurement sensitivity and is beneficial for the measurement. Conductivity measurement experiments of KCl solutions are also conducted. The experimental results are in agreement with the simulation results. According to the results of both simulation and experiments, the design criterion is concluded. Considering the practical applications, setting the electrode length as 5–10 times of the pipe inner diameter and the electrode angle as 120–160° is recommended for the design of C^4^D sensors with radial configuration.

This work focuses on the investigation of the effects of electrode geometry on the performance of the sensor and the determination of the optimal electrode geometry. Except for this aspect, many other factors, such as the effects of the coupling capacitance, the stray capacitance, the material of the pipe and so on, are also worth considering for the research or application of radial configuration. These can be the basis for further work upon the radial configuration.

## Figures and Tables

**Table 1:** The properties of the model components.

Components	Materials	Conductivity (mS/cm)	Relative Permittivity
pipe wall	quartz glass	0	4.2
electrodes	copper	$5\times 10^{8}$	
external air	air	0	1
fluid	solution	0.04~10	78

**Table 2:** The geometry parameters of all the C^4^D sensors.

	Electrode Length L (mm)	L/d	Electrode Angle θ (°)
Sensor 1	15.00	1.93	120
Sensor 2	40.00	5.15	120
Sensor 3	60.00	7.73	120
Sensor 4	40.00	5.15	80
Sensor 5	40.00	5.15	160

## Data Availability

The data presented in this study are available on request from the corresponding author. The data are not publicly available due to protection of intellectual property.
